# Caring for the Stonewall Generation: Assessment of Staff Training for Transgender Persons Living in Long Term Care

**DOI:** 10.1093/geroni/igab046.3147

**Published:** 2021-12-17

**Authors:** Sara English

**Affiliations:** University of South Carolina, Blair, South Carolina, United States

## Abstract

Transgender persons who came of age in the late 1960s are considered LGBTQ+ elders - The Stonewall Generation. These persons experience unique bio-psychosocial challenges, often complicated by a history of a lack of access to good medical care and social supports. Discrimination and bias can influence the provision of care and the protection of privacy for transgender or gender non-conforming persons. Staff training is essential to provide ethical care for aging trans persons who require residential care. This presentation examines current staff training modules of 100 Long Term Care (LTC) facilities, assessing training needs to provide affirming, culturally competent, and ethical care for sexual and gender minorities.

Keywords: cultural competence; Long Term Care; staff training; transgender

